# A Comprehensive Analysis of Hyperbolical Fluids in Modified Gravity

**DOI:** 10.3390/e24020150

**Published:** 2022-01-19

**Authors:** Z. Yousaf, M. Z. Bhatti, Maxim Khlopov, H. Asad

**Affiliations:** 1 Department of Mathematics, Quaid-i-Azam Campus, University of the Punjab, Lahore 54590, Pakistan; mzaeem.math@pu.edu.pk (M.Z.B.); hamnaasad2596@gmail.com (H.A.); 2 Institute of Physics, Southern Federal University, 194 Stachki, 344090 Rostov-on-Donu, Russia; khlopov@apc.in2p3.fr; 3 CNRS, Astroparticule et Cosmologie, Universite de Paris, F-75013 Paris, France; 4 Institute of Nuclear Physics and Engineering, National Research Nuclear University MEPhI, 115409 Moscow, Russia

**Keywords:** mathematical cosmology, gravitation, anisotropy, mathematical techniques

## Abstract

This paper is devoted to understanding a few characteristics of static irrotational matter content that assumes hyperbolical symmetry. For this purpose, we use metric f(R) gravity to carry out our analysis. It is noticed that the matter distribution cannot fill the region close to the center of symmetry, thereby implying the existence of an empty core. Moreover, the evaluation of the effective energy density reveals that it is inevitably negative, which could have utmost relevance in understanding various quantum field events. To derive the structure scalars, we perform the orthogonal splitting of the Riemann tensor in this modified gravity. Few relationships among matter variables and both Tolman and Misner Sharp are determined. Through two generating functions, some hyperbolically symmetric cosmological models, as well as their physical interpretations, are studied. To delve deeply into the role of f(R) terms, the model of the less-complex relativistic system of Einstein gravity is presented.

## 1. Introduction

General Relativity (GR) has emerged as one of the best theories to explain various cosmic issues, such as gravitational redshift, orbital precession, light deflection, gravitational lensing, black hole prediction, and frame transition of spinning bodies in spacetime [1]. In spite of this fact, various theoretical physicists suggested that this theory needs to modify for a better understanding of our cosmos. Our understanding towards universe formation as well as its ultimate fate is mystifying, and thus requires more explanation. It is also well-known that astrophysicists and cosmologists discovered some pieces of evidence that paint a surprising accelerating expanding picture of our cosmos. As a consequence, the idea of modified theories of gravities has become among the most attractive approaches to explain these queries. Therefore, f(R) gravity theory was introduced by modifying the gravitational field of GR [2]. In this gravitational model, the Ricci scalar *R*, which appears in the GR action integral, was replaced with its generic function. Over the last few years, those alternative gravity theories that are based on a f(R) Lagrangian gained significant emphasis [3,4,5]. Few researchers [6,7,8,9,10,11,12] presented their analysis based on f(R) theory. They proposed a cosmic model that could be considerably useful to explain unknown universe matter components and inflation.

In order to deduce equations of motion from the f(R) action, there are three applicable ways of variational principles. These are named as metric formalism (metric f(R) gravity), Palatini formalism (Palatini f(R) gravity) and metric-affine formalism (metric-affine f(R) gravity). In metric formalism [13,14], the action varies with respect to the metric tensor and the metric is minimally coupled to the matter. This approach leads to second order differential equation. In Palatini formalism [15,16,17], the action varies with regard to both the metric tensor and Christoffel symbols. The matter action in it does not only depend upon Christoffel symbols. The f(R) theories are conserved and hence to show that $T_{\pi \beta }$ is divergence-free, one can utilize the typical arguments based on the action’s invariance under diffeomorphisms of the spacetime manifold. It is all about the transformation of the coordinates ($x^{\beta }\to x^{{}^{\prime }\beta }=x^{\beta }+\eta^{\beta }$), with the vanishing field on the considered boundary of spacetime region. In all of this process, physics remains conserved [18]. For a concise discussion of metric and Palatini f(R) gravity see [19]. Also look at [20] for a comprehensive examination of all variations of f(R) gravity and other alternative theories of gravity.

Cavity is an astronomical object with an apparent hollow structure, such as a large hole on the surface of a molecular cloud generated by the ultraviolet photons of a big star. Cavity forms around condensation, which gradually spreads and deepens over time, due to the conservation of mass. An energy depletion of cellular structures produces condensation and a cavity around it. In some situations, a narrow shell of matter with a density significantly higher than the mean cosmological value surrounds the cavity. However, there are initial density profile options that lead to the creation of deep voids. Large-scale holes in galaxy distribution can be used to identify these “walled-in" cavities. Astronomers were looking through 3D maps of the shapes and sizes of surrounding molecular clouds when they made the latest finding.

Occhionero [21] examined the evolution of inhomogeneities using Tolmann models that asymptotically become uniform Friedmann models, and demonstrated that cavities arise around condensations in those circumstances. Speaking briefly, they have discussed the formation of cavities around cosmological condensation. Hausman et al. [22] investigated the genesis and nonlinear evolution of those spherically symmetric objects that occupy negative density. They deduced some results about the structural evolution of the cavities inside the galaxies. Occhionero et al. [23] offered an algorithm that describes non-linear growth of cavities and ridges in the Hubble flow. The main idealization in that was the pressureless spherical symmetry. Moreover, they constructed the models on the basis of two parameters that relate the initial perturbation’s amplitude with the shape function of the surrounding cavity (or mass ridges). Goryachev et al. [24] demonstrated that detecting hypothesized particles such as paraphotons and axions, which make up the universe’s dark sector, can be reduced to detecting extremely weak linkages or couplings between cavities and modes.

Harrison [25] was the first to examine a solution to the Einstein equations of the particular form, which was determined by the hyperbolic symmetry and it has since been the topic of investigation in several contexts [26,27,28,29,30,31,32,33,34]. Herrera et al. [35] have done a detailed investigation on static fluid distributions with hyperbolical symmetry in the framework of GR. Furthermore, it is discovered that the fluid distribution is unable to fill the region around the symmetry center.

Bhatti et al. continued the Herrera’s work under the influence of electromagnetic force [36] and modified gravity [37] in order to provide a coherent analysis of hyperbolically symmetric static sources. They investigated the physical effects of such a force on the substantial characteristics of the hyperbolically symmetric spacetime. Herrera et al. [38] examined the generic features of dissipative fluid distributions with hyperbolical symmetry in GR. They discovered some intriguing thermodynamical features of these fluids by assuming a causal transport equation. Along with this, the vanishing complexity factor models are presented in the quasi–homologous regime. Lobo and Mimiso [39] used static and pseudo-spherically symmetric spacetime tunnels to produce solutions of a specific class. They also examined the physical elements of these solutions and looked into the concept of tunnels in hyperbolic spacetime. Herrera et al. [40] analyzed the fluid distributions with hyperbolic symmetry, which are similar to Lemaitre–Tolman–Bondi (LTB) solutions, when the system experienced geodesic, non-conformally flat and shearing limits. They examined the pure dust models as well as the dissipative models with anisotropic pressure. Moreover, they deduced the noteworthy fact that all solutions satisfying the vanishing complexity factor criterion are non-dissipative and satisfy the stiff equation of state.

This article is the continuation of the analysis conducted by Herrera et al. [35] in the metric f(R) gravity. In order to achieve that, we used modified field equations to determine the effects of modified gravity on hyperbolically symmetric self gravitating objects. The following is a description of how we systematized our paper. In Section 1, the basic formalism of f(R) gravity as well as interpretation of effective matter are presented. In Section 2, field equations are evaluated for the aforementioned gravity. For our system, the Riemann tensor, Weyl tensor, and active gravitational mass are derived in Section 3. The orthogonal splitting of the curvature tensor is evaluated in Section 4. In Section 5, various hyperbolically symmetric solutions accompanying two generating functions are examined. All the outcomes are summarized in Section 6.

## 2. Basic Formalism of the f(R) Theory

The action for metric f(R) gravity is

(1) $$S_{f(R)}=\frac{1}{2\kappa }\int d^{4}x\sqrt{-g}f\left(R\right)+S^{m},$$

where $S^{m}$ indicates the action’s matter part and κ is the coupling constant whose value is 8π in our case, as the normalized units G=c=1 are taken into consideration. The action varies in the metric formalism [41,42] with regard to the metric $g^{\pi \beta }$. The field equations that arises after the implementation of aforementioned condition are as follows

(2) $$f_{R}R_{\pi \beta }-\frac{f\left(R\right)g_{\pi \beta }}{2}=\nabla_{\pi }\nabla_{\beta }f_{R}-g_{\pi \beta }□f_{R}+\kappa T_{\pi \beta },$$

where the covariant derivative of $g^{\pi \beta }$ is represented by $\nabla_{\pi }$. The d’Alembert operator is symbolized and defined as $□=g^{\gamma \beta }\nabla_{\gamma }\nabla_{\beta }$. Equation (2) generates

$$f_{R}R+3□f_{R}-2f\left(R\right)=\kappa T.$$

On the other hand, the trace equation of GR is just the algebraic equation R=−κT, indicating that $f_{R}$ is a propagating degree of freedom. Equation (2) can be expressed as

(3) $$G_{\pi \beta }=\kappa T_{\pi \beta }^{\left(eff\right)}=\frac{\kappa }{f_{R}}\left(T_{\pi \beta }^{m}+T_{\pi \beta }^{D}\right),$$

where $T_{\pi \beta }^{D}$ and $T_{\pi \beta }^{m}$ are

(4) $$\begin{array}{cc} T_{\pi \beta }^{m}= & \left(\mu +P\right)V_{\pi }V_{\beta }-Pg_{\pi \beta }+\Pi_{\pi \beta },\\ T_{\pi \beta }^{D}= & \frac{1}{\kappa }\left(\frac{f\left(R\right)-Rf_{R}}{2}g_{\pi \beta }+\nabla_{\pi }\nabla_{\beta }f_{R}-g_{\pi \beta }□f_{R}\right), \end{array}$$

where *P* and $\Pi_{\alpha \nu }$ represent the anisotropic pressure and anisotropic tensor of the fluid. The vector $V_{\mu }$ is a fluid’s four velocity and μ is the corresponding energy density of the matter. They are described as follows

$$\begin{array}{ccc} P & = & \frac{P_{r}+2P_{⊥}}{3},\;\Pi_{\pi \beta }=\Pi \left(K_{\pi }K_{\beta }+\frac{h_{\pi \beta }}{3}\right),\\ h_{\pi \beta } & = & g_{\pi \beta }-V_{\beta }V_{\pi },\;\Pi =P_{r}-P_{⊥}. \end{array}$$

where $P_{⊥}$ and $P_{r}$ are the tangential and radial pressure components and $K_{\mu }$ is a four vector. For Equations (3) and (4) formulates $T_{\pi \beta }^{\left(eff\right)}$ as

(5) $$T_{\pi \beta }^{\left(eff\right)}=\frac{1}{f_{R}}\left\{T_{\pi \beta }^{m}+\frac{1}{\kappa }\left(\frac{f\left(R\right)-Rf_{R}}{2}g_{\pi \beta }+\nabla_{\pi }\nabla_{\beta }f_{R}-g_{\pi \beta }□f_{R}\right)\right\}.$$

## 3. Modified Field Equations

The state variables and equations required to describe a static self-gravitating locally anisotropic fluid admitting the four Killing vectors will be discussed in this section. To achieve this goal, we have taken the hyperbolically symmetric static fluid, which is enclosed from the outer surface. This boundary can be mathematically represented with the equation $r=r_{\Sigma^{e}}=$ constant. However, the fluid distribution cannot fill the central region, therefore we may suppose that this region is portrayed by an empty vacuole, suggesting that the fluid distribution is likewise restricted from the inside by a surface and is expressed with the equation $r=r_{\Sigma^{i}}=$ constant. We model our system with the line element described as below

(6) $$ds^{2}=e^{\lambda (r)}dt^{2}-e^{\nu (r)}dr^{2}-r^{2}d\theta^{2}-r^{2}\sinh^{2}\theta d\phi^{2}.$$

W obtain the following modified field equations with the use of Equation (3) as well as the line element (6) as

(7) $$\begin{array}{cc} 8\pi \mu^{\left(eff\right)} & =-\frac{1+e^{-\nu }}{r^{2}}+\frac{\lambda^{\prime }e^{-\nu }}{r}, \end{array}$$

(8) $$\begin{array}{cc} 8\pi P_{r}^{\left(eff\right)} & =\frac{1+e^{-\nu }}{r^{2}}+\frac{\nu^{\prime }e^{-\nu }}{r}, \end{array}$$

(9) $$\begin{array}{cc} 8\pi P_{⊥}^{\left(eff\right)} & =\frac{e^{-\nu }}{2}\left(\lambda^{\prime \prime }-\frac{\lambda^{\prime }\nu^{\prime }}{2}+\frac{\lambda^{\prime 2}}{2}-\frac{\nu^{\prime }}{r}+\frac{\lambda^{\prime }}{r}\right), \end{array}$$

where $\mu^{\left(eff\right)},\;P_{r}^{\left(eff\right)}$, and $P_{⊥}^{\left(eff\right)}$ denote effective matter density and pressures in various directions, respectively, and prime denotes derivatives relative to *r*. Their values are defined in the Appendix A. It is noteworthy to accentuate the differences between these equations and those mentioned in [43] by taking into account the spherically symmetric case. Moreover, the f(R) theory is conserved, so its conservation equation is evaluated as follows

(10) $$\frac{\partial P_{r}^{\left(eff\right)}}{\partial r}+\frac{\lambda^{\prime }}{2}\left(\mu^{\left(eff\right)}+P_{r}^{\left(eff\right)}\right)+\frac{2\Pi^{\left(eff\right)}}{r}=0,$$

where $P_{r}^{\left(eff\right)}=P_{⊥}^{\left(eff\right)}+\Pi^{\left(eff\right)}$. The distribution of hyperbolically symmetric fluid has mass function m(r) as

(11) $$m\left(r\right)=\frac{r}{2}\left(1+e^{-\nu }\right).$$

After substituting Equation (11) into Equation (7), the value of m(r) becomes

(12) $$m\left(r\right)=-4\pi \int_{0}^{r}\mu^{\left(eff\right)}r^{2}dr.$$

The mass *m* and the effective density $\mu^{\left(eff\right)}$ should be regarded as positive and negative quantities, respectively, according to Equations (11) and (12). As previously discovered in [33], the weak energy requirement is therefore disobeyed. Some intriguing statements on the physical significance of Equation (12) have been mentioned in [35]. Finally the mass function turns out to be

(13) $$m\left(r\right)=4\pi \int_{r_{min}}^{r}\left|\mu^{\left(eff\right)}\right|r^{2}dr.$$

Equation (13) is obtained by substituting $-|\mu^{\left(eff\right)}|$ for $\mu^{\left(eff\right)}$. Utilizing Equations (9) and (11) we achieve

(14) $$\lambda^{\prime }=\left\{\frac{8\pi P_{r}^{\left(eff\right)}r^{3}-2m}{r(2m-r)}\right\}.$$

We calculate the hydrostatic equilibrium condition by substituting the obtained value of $\lambda^{\prime }$ from Equation (14) into Equation (10), which is as follows

(15) $$\frac{\partial P_{r}^{\left(eff\right)}}{\partial r}+\left(\frac{4\pi P_{r}^{\left(eff\right)}r^{3}-m}{r(2m-r)}\right)(P_{r}^{\left(eff\right)}-|\mu^{\left(eff\right)}\left|\right)+\frac{2\Pi^{\left(eff\right)}}{r}=0.$$

In [35], a complete discussion on the physical influence of Equation (15) has also been given.

## 4. Intrinsic Curvature and Conformal Tensor

On the basis of the Riemann tensor, Ricci tensor, and Ricci scalar [44], the curvature of spacetime can be measured intrinsically. These three curvature tensors are used to illustrate the Conformal tensor [45], which is written as follows

(16) $$R_{\eta \rho \beta }^{\pi }=C_{\eta \rho \beta }^{\pi }+\frac{1}{2}R_{\rho }^{\pi }g_{\eta \beta }+\frac{1}{2}R_{\eta \rho }\delta_{\beta }^{\pi }+\frac{1}{2}R_{\eta \beta }\delta_{\rho }^{\pi }-\frac{1}{2}R_{\beta }^{\pi }g_{\eta \rho }-\frac{1}{6}R\left(\delta_{\rho }^{\pi }g_{\eta \beta }-g_{\eta \rho }\delta_{\beta }^{\pi }\right).$$

In our scenario, the conformal tensor can be seen by looking at its electric portion only (as the magnetic part becomes zero)

$$C_{\xi \pi \mu \lambda }=E^{\beta \delta }V^{\rho }V^{\gamma }\left(g_{\xi \pi \rho \beta }g_{\mu \lambda \gamma \delta }-\eta_{\xi \pi \rho \beta }\eta_{\mu \lambda \gamma \delta }\right),$$

where

$$g_{\xi \pi \rho \beta }=g_{\xi \rho }g_{\pi \beta }-g_{\xi \beta }g_{\pi \rho },$$

where $\eta_{\xi \pi \rho \beta }$ depicts the Levi-Civita tensor. In favor of our metric, the electric component of Conformal tensor, i.e., $E_{\mu \beta }$ is defined as

(17) $$E_{\pi \beta }=\varepsilon \left(K_{\pi }K_{\beta }+\frac{1}{3}h_{\pi \beta }\right),$$

where the conformal scalar is indicated by ε. The electric portion of the conformal tensor has physical consequences that coincide with tidal forces. It uses an appropriately rescaled curvature on the hyperboloid D to show how neighboring geodesics break apart from each other when approaching spatial infinity. The ε is calculated in this case as

(18) $$\begin{array}{cc} \varepsilon & =-\frac{\lambda^{\prime \prime }e^{-\nu }}{4}+\frac{\nu^{\prime }\lambda^{\prime }e^{-\nu }}{8}-\frac{\lambda^{\prime 2}e^{-\nu }}{8}+\frac{\lambda^{\prime }e^{-\nu }}{4r}-\frac{\nu^{\prime }e^{-\nu }}{4r}-\frac{e^{-\nu }}{2r^{2}}-\frac{1}{2r^{2}}. \end{array}$$

Through Equations (7), (9), (11) and (18) we evaluate

(19) $$\frac{3m}{r^{3}}+\varepsilon =4\pi \left|\mu^{\left(eff\right)}\right|+4\pi \Pi^{\left(eff\right)}.$$

Taking into account Equation (12) along with the derivative of Equation (19) corresponds to *r* produce

(20) $$\varepsilon -4\pi \Pi^{\left(eff\right)}=\frac{4\pi }{r^{3}}\int_{0}^{r}\frac{\partial |\mu^{\left(eff\right)}|}{\partial r}r^{3}dr.$$

On the behalf of Equation (20) one can say that the effective anisotropic pressure tensor and the inhomogeneity of effective matter density can be used to express the conformal scalar. Computing Equation (20) in Equation (19) causes it to assume the form

(21) $$m+\frac{4\pi }{3}\int_{0}^{r}\frac{\partial |\mu^{\left(eff\right)}|}{\partial r}r^{3}dr=\frac{4\pi |\mu^{\left(eff\right)}|r^{3}}{3}.$$

Equation (21) illustrates that the homogeneous effective energy density as the sum of the inhomogeneity induced in the effective energy density and the mass function.

### Tolman Mass

Several years back, Tolman [46] described a general formula to study the mass function of a fluid sphere. The active gravitational mass for every static hyperbolically symmetric fluid distribution, is then formalized as

(22) $$m_{T}=\int_{0}^{2\pi }\int_{0}^{\pi }\int_{0}^{r}r^{2}e^{\frac{\lambda +\nu }{2}}\sinh\theta \left(T_{0}^{0(eff)}-T_{1}^{1(eff)}-2T_{2}^{2(eff)}\right)d\tilde{r}d\theta d\phi ,$$

where the standard stress-energy tensor components are depicted by $T_{0}^{0(eff)},T_{1}^{1(eff)}$ and $T_{2}^{2(eff)}$. Computing their respective values in Equation (22), we achieve

(23) $$m_{T}=2\pi \left(cosh\pi -1\right)\int_{0}^{r}e^{\frac{\nu +\lambda }{2}}{\tilde{r}}^{2}\left(-|\mu^{\left(eff\right)}|+P_{r}^{\left(eff\right)}+2P_{⊥}^{\left(eff\right)}\right)d\tilde{r}.$$

Integration of Equation (22) and utilization of modified field Equations (7)–(9), respectively, generate

(24) $$m_{T}=\lambda^{\prime }r^{2}e^{\frac{\lambda -\nu }{2}}\frac{\cosh\pi -1}{4}.$$

Utilizing Equation (24) with that of Equation (14) produces

(25) $$m_{T}=\frac{\cosh\pi -1}{2}\left(4\pi P_{r}^{\left(eff\right)}r^{3}-m\right)e^{\frac{\lambda +\nu }{2}}.$$

The typical physical analysis of the Tolmann mass ($m_{T}$) can be studied through Equations (10), (15), (24) and (25). It can therefore be seen that, if $4\pi P_{r}^{\left(eff\right)}r^{3}<m$ then $m_{T}$ becomes negative, thereby suggesting the repulsive nature of the spacetime. The four acceleration $a_{\pi }$ is defined as

(26) $$a_{\pi }=aK_{\pi },$$

where $a=\frac{\lambda^{\prime }e^{\frac{-\nu }{2}}}{2}$. Substituting the value of $\lambda^{\prime }$ from Equation (24), Equation (26) turns into

(27) $$a=\frac{2m_{T}e^{\frac{-\lambda }{2}}}{r^{2}\left(\cosh\pi -1\right)}.$$

It is possible to achieve the radially inward flow of four accelerations, if we take $4\pi P_{r}^{\left(eff\right)}r^{3}<m$, thus making $m_{T}$ as a negative quantity. This leads towards the repulsive character of gravitational force. Afterwards, utilizing Equation (25) with the *r*-derivative of Equation (22), we obtain

(28) $$m_{T}^{\prime }-\frac{3m_{T}}{r}=-\left(\frac{\cosh\pi -1}{2}\right)r^{2}e^{\frac{\nu +\lambda }{2}}\left(\varepsilon +4\pi \left[\frac{\Pi }{f_{R}}+e^{-\nu }f_{R}^{\prime \prime }-\frac{e^{-\nu }f_{R}^{\prime }\nu^{\prime }}{2}-\frac{f_{R}^{\prime }e^{-\nu }}{r}\right]\right).$$

Integration of Equation (28) gives

(29) $$\begin{array}{cc} m_{T}-{\left(m_{T}\right)}_{\Sigma_{e}}\left(\frac{r^{3}}{r_{\Sigma^{e}}^{3}}\right) & =\left(\frac{\cosh\pi -1}{2}\right)r^{3}\int_{r}^{r_{\Sigma^{e}}}\frac{e^{\frac{\nu +\lambda }{2}}}{\tilde{r}}(\varepsilon +4\pi [\frac{\Pi }{f_{R}}+e^{-\nu }f_{R}^{\prime \prime }\\ & -\frac{e^{-\nu }f_{R}^{\prime }\nu^{\prime }}{2}-\frac{f_{R}^{\prime }e^{-\nu }}{r}])d\tilde{r}. \end{array}$$

Computing Equation (20) in Equation (28) to achieve

(30) $$\begin{array}{cc} m_{T}-{\left(m_{T}\right)}_{\Sigma^{e}}\left(\frac{r^{3}}{r_{\Sigma^{e}}^{3}}\right) & =\left(\frac{\cosh\pi -1}{2}\right)r^{3}\int_{r}^{r_{\Sigma^{e}}}\frac{e^{\frac{\nu +\lambda }{2}}}{\tilde{r}}[\frac{4\pi }{{\tilde{r}}^{3}}\int_{0}^{r}\frac{\partial |\mu^{\left(eff\right)}|}{\partial r}{\tilde{r}}^{3}d\tilde{r}\\ & +8\pi \left(\frac{\Pi }{f_{R}}+e^{-\nu }f_{R}^{\prime \prime }-\frac{e^{-\nu }f_{R}^{\prime }\nu^{\prime }}{2}-\frac{f_{R}^{\prime }e^{-\nu }}{r}\right)]d\tilde{r}. \end{array}$$

With the inclusion of effective matter terms, the conclusion of Equation (30) is the same as that determined in Equation (54) in the [35].

## 5. Orthogonal Splitting of Curvature Tensors

On the basis of the orthogonal splitting approach of the Riemann tensor studied by Bel [47] and followed by [48,49,50,51,52,53], we shall calculate structure scalars in metric f(R) gravity. We shall use terminologies with minor changes as that utilized in [48]. Through orthogonal splitting, we end up with the following three tensors

$$\begin{array}{ccc} & & Y_{\pi \beta }=R_{\pi \xi \beta \delta }u^{\xi }u^{\delta },\\ & & Z_{\pi \beta }{=}^{*}R_{\pi \xi \beta \delta }u^{\xi }u^{\delta }=\frac{1}{2}\eta_{\pi \xi \epsilon \rho }R_{\beta \delta }^{\epsilon \rho }u^{\xi }u^{\delta },\\ & & X_{\pi \beta }{=}^{*}R_{\pi \xi \beta \delta }^{*}u^{\xi }u^{\delta }=\frac{1}{2}\eta_{\pi \xi }^{\epsilon \rho }R_{\epsilon \rho \beta \delta }^{*}u^{\xi }u^{\delta }, \end{array}$$

where * depicts the dual tensor and hence $R_{\pi \xi \beta \delta }^{*}$ is expressed as

$$R_{\pi \beta \xi \delta }^{*}=\frac{1}{2}\eta_{\epsilon \omega \xi \delta }R_{\pi \beta }^{\epsilon \omega }.$$

Through modified field equations, Equation (16) gives

(31) $$\begin{array}{c} R_{\beta \gamma }^{\mu \xi }=C_{\beta \gamma }^{\mu \xi }+16\pi {\left.T\right.}_{[\beta }^{[\mu }\delta_{\gamma ]}^{\xi ]}+8\pi \left.T\right.\left(\frac{1}{3}\delta_{[\beta }^{\mu }\delta_{\gamma ]}^{\xi }-\delta_{[\beta }^{[\mu }\delta_{\gamma ]}^{\xi ]}\right). \end{array}$$

When we substitute Equation (4) back into Equation (31), we get

$$R_{\beta \gamma }^{\mu \xi }=R_{(I)\beta \gamma }^{\mu \xi }+R_{(II)\beta \gamma }^{\mu \xi }+R_{(III)\beta \gamma }^{\mu \xi },$$

where

(32) $$\begin{array}{cc} R_{(I)\beta \gamma }^{\mu \xi } & =\frac{16\pi }{f_{R}}\left(\mu V^{[\mu }V_{[\beta }\delta_{\gamma ]}^{\xi ]}+Ph_{[\gamma }^{[\mu }\delta_{\beta ]}^{\xi ]}\right)+\frac{8\pi }{f_{R}}\left[\left(\mu -3P\right)+2\left(f-Rf_{R}\right)-3\nabla_{\xi }\nabla^{\xi }f_{R}\right]\\ & \times \left(\frac{1}{3}\delta_{[\beta }^{\mu }\delta_{\gamma ]}^{\xi }-\delta_{[\beta }^{[\mu }\delta_{\gamma ]}^{\xi ]}\right), \end{array}$$

(33) $$\begin{array}{cc} R_{(II)\beta \gamma }^{\mu \xi } & =\frac{16\pi }{f_{R}}\Pi_{[\beta }^{[\mu }\delta_{\gamma ]}^{\xi ]}+\frac{2}{f_{R}}[\left(f-Rf_{R}\right)\left(\delta_{\beta }^{\mu }\delta_{\gamma }^{\xi }-\delta_{\gamma }^{\mu }\delta_{\beta }^{\xi }\right)+(\nabla^{\mu }\nabla_{\beta }\delta_{\gamma }^{\xi }\\ & -\nabla^{\mu }\nabla_{\gamma }\delta_{\beta }^{\xi }-\nabla^{\xi }\nabla_{\beta }\delta_{\gamma }^{\mu }+\nabla^{\xi }\nabla_{\gamma }\delta_{\beta }^{\mu })f_{R}+2\left(\delta_{\gamma }^{\mu }\delta_{\beta }^{\xi }-\delta_{\beta }^{\mu }\delta_{\gamma }^{\xi }\right)\nabla^{\rho }\nabla_{\rho }f_{R}], \end{array}$$

(34) $$\begin{array}{cc} R_{(III)\beta \gamma }^{\mu \xi } & =4V^{[\mu }V_{[\beta }E_{\gamma ]}^{\xi ]}-\epsilon_{\delta }^{\mu \xi }\epsilon_{\pi \beta \gamma }E^{\delta \pi }. \end{array}$$

In order to calculate three tensors $\left(Y_{\pi \beta },\;Z_{\pi \beta },X_{\pi \beta }\right)$ in terms of the structural parameters, Equations (32)–(34) give

(35) $$\begin{array}{cc} Y_{\pi \beta } & =E_{\pi \beta }+\frac{4\pi }{f_{R}}\Pi_{\pi \beta }+\frac{4\pi h_{\pi \beta }}{3f_{R}}\left(\mu +3P\right)+\frac{1}{6}\left(Rf_{R}-f\right)+\frac{1}{2f_{R}}[\nabla_{\pi }\nabla_{\beta }f_{R}\\ & -V_{\pi }V^{\gamma }\nabla_{\beta }\nabla_{\gamma }f_{R}-V_{\beta }V^{\xi }\nabla_{\pi }\nabla^{\xi }f_{R}+g_{\pi \beta }V_{\xi }V^{\gamma }\nabla^{\xi }\nabla_{\gamma }f_{R}], \end{array}$$

(36) $$\begin{array}{cc} X_{\pi \beta } & =\frac{8\pi }{3f_{R}}P-\frac{2\pi }{f_{R}}\left(|\mu |+3P\right)+\frac{1}{4f_{R}}\nabla^{\rho }\nabla_{\rho }f_{R}h_{\pi \beta }+\frac{4\pi }{f_{R}}\Pi_{\pi \beta }+\frac{1}{2f_{R}}\nabla^{\mu }\nabla_{\sigma }\epsilon_{\pi }^{\sigma \gamma }\epsilon_{\mu \gamma \beta }\\ & -E_{\pi \beta }, \end{array}$$

(37) $$\begin{array}{cc} Z_{\pi \beta } & =-\frac{1}{2f_{R}}\epsilon_{\mu \pi \beta }V^{\gamma }\nabla^{\mu }\nabla_{\gamma }. \end{array}$$

The aforementioned tensors can be decomposed into their trace and trace-free portions in the following way

$$\begin{array}{cc} X_{\pi \beta } & =\frac{h_{\pi \beta }}{3}X_{T}+\left(K_{\pi }K_{\beta }+\frac{h_{\pi \beta }}{3}\right)X_{TF},\\ Y_{\pi \beta } & =\frac{h_{\pi \beta }}{3}Y_{T}+\left(K_{\pi }K_{\beta }+\frac{h_{\pi \beta }}{3}\right)Y_{TF}. \end{array}$$

Using the trace and trace-free sections of both tensors, the following results are produced

(38) $$\begin{array}{cc} X_{T} & =-\frac{6\pi }{f_{R}}\left(|\mu \right|-\frac{5P}{3})+\frac{3}{4}\nabla^{\rho }\nabla_{\rho }f_{R}-\frac{1}{f_{R}}\left(\nabla^{\mu }\nabla_{\sigma }h_{\mu }^{\sigma }\right), \end{array}$$

(39) $$\begin{array}{cc} X_{TF} & =\frac{4\pi \Pi }{f_{R}}-\varepsilon +\xi^{DR}, \end{array}$$

(40) $$\begin{array}{cc} Y_{T} & =\frac{4\pi }{f_{R}}\left(-|\mu |+3P\right)+\frac{1}{2f_{R}}\left(Rf_{R}-f\right)+\frac{1}{2f_{R}}[□f_{R}-g^{\pi \beta }V_{\pi }V^{\gamma }\nabla_{\beta }\nabla_{\gamma }f_{R}\\ \; & -g^{\pi \beta }V_{\xi }V_{\beta }\nabla^{\xi }\nabla_{\pi }f_{R}+4V_{\xi }V^{\gamma }\nabla^{\xi }\nabla_{\gamma }f_{R}], \end{array}$$

(41) $$\begin{array}{cc} Y_{TF} & =\varepsilon +\frac{4\pi \Pi }{f_{R}}-\frac{1}{6f_{R}\left(K_{\pi }K_{\beta }+\frac{h_{\pi \beta }}{3}\right)}\left(h_{\pi \beta }V_{\xi }V^{\gamma }\nabla^{\xi }\nabla_{\gamma }f_{R}\right), \end{array}$$

where $\xi^{DR}$ is given in Appendix A. We now use Equation (20) in the expressions of $X_{TF}$ and $Y_{TF}$ to generate

(42) $$\begin{array}{cc} X_{TF} & =\frac{4\pi \Pi }{f_{R}}-\frac{4\pi }{r^{3}}\int_{0}^{r}{\tilde{r}}^{3}\frac{\partial |\mu^{\left(eff\right)}|}{\partial r}d\tilde{r}-4\pi \Pi^{\left(eff\right)}+\xi^{DR}, \end{array}$$

(43) $$\begin{array}{cc} Y_{TF} & =4\pi \Pi^{\left(eff\right)}+\frac{4\pi }{r^{3}}\int_{0}^{r}{\tilde{r}}^{3}\frac{\partial |\mu^{\left(eff\right)}|}{\partial r}d\tilde{r}+\frac{4\pi \Pi }{f_{R}}-\frac{4\pi }{f_{R}\left(K_{\pi }K_{\beta }+\frac{h_{\pi \beta }}{3}\right)}\left(h_{\pi \beta }V_{\xi }V^{\gamma }\nabla^{\xi }\nabla_{\gamma }f_{R}\right), \end{array}$$

which gives the anisotropic tensor from the sum of $X_{TF}$ and $Y_{TF}$ as

$$8\pi \Pi =f_{R}\left(X_{TF}+Y_{TF}-\xi^{DR}\right)+\frac{1}{6(K_{\pi }K_{\beta }+\frac{h_{\pi \beta }}{3})}\left(h_{\pi \beta }V_{\xi }V^{\gamma }\nabla^{\xi }\nabla_{\gamma }f_{R}\right).$$

Returning to Equations (23) and (30), we can use Equations (40) and (43) to establish the physical relevance of $Y_{T}$ and $Y_{TF}$ as

(44) $$\begin{array}{cc} m_{T} & ={\left(m_{T}\right)}_{\Sigma^{e}}{\left(\frac{r}{r_{\Sigma e}}\right)}^{3}+\left(\frac{cosh\pi -1}{2}\right)r^{3}\int_{r}^{r_{\Sigma^{e}}}\frac{e^{\frac{\nu +\lambda }{2}}}{\tilde{r}}(Y_{TF}-4\pi \Pi^{\left(eff\right)}\\ & -\frac{4\pi \Pi }{f_{R}}+\frac{1}{6f_{R}\left(K_{\pi }K_{\beta }+\frac{h_{\pi \beta }}{3}\right)}\left(h_{\pi \beta }V_{\xi }V^{\gamma }\nabla^{\xi }\nabla_{\gamma }f_{R}\right)d\tilde{r}, \end{array}$$

(45) $$\begin{array}{cc} m_{T} & =\frac{cosh\pi -1}{2}\int_{0}^{r}{\tilde{r}}^{2}e^{(\nu +\lambda )/2}[Y_{T}f_{R}+\frac{1}{f_{R}}(-Rf_{R}+f-□f_{R}\\ & +g^{\pi \beta }V_{\pi }V^{\gamma }\nabla_{\beta }\nabla_{\gamma }f_{R}+g^{\pi \beta }V_{\xi }V_{\beta }\nabla^{\xi }\nabla_{\pi }f_{R}-4V_{\xi }V^{\gamma }\nabla^{\xi }\nabla_{\gamma }f_{R})]d\tilde{r}. \end{array}$$

The influence of density inhomogeneity and pressure anisotropy on the Tolman mass has been taken into consideration by $Y_{TF}$. Alternatively, $Y_{TF}$ illustrates how these two variables change the value of the Tolman mass, comparable to its value for the homogeneous isotropic fluid. This sparked the idea of complexity, which was discussed in [54,55,56,57,58,59,60].

## 6. Hyperbolically Symmetric Static Solutions

With the help of two generating functions, a general framework for expressing any static hyperbolically symmetric solutions will be presented in this section. Equations (8) and (9) produce

(46) $$8\pi \left(P_{r}^{\left(eff\right)}-P_{⊥}^{\left(eff\right)}\right)=\frac{1+e^{-\nu }}{r^{2}}-\frac{e^{-\nu }}{2}\left(\lambda^{\prime \prime }+\frac{\lambda^{\prime 2}}{2}-\frac{\lambda^{\prime }\nu^{\prime }}{2}-\frac{\lambda^{\prime }}{r}-\frac{\nu^{\prime }}{r}\right).$$

The involvement of the auxiliary functions, i.e., $\frac{\lambda^{\prime }}{2}=z^{*}-\frac{1}{r}$ and $\tilde{y}=e^{-\nu }$ in Equation (46) modify it into

(47) $$\begin{array}{c} {\tilde{y}}^{\prime }+\tilde{y}\left[\frac{4}{r^{2}z^{*}}+2z^{*}+\frac{2z^{{*}^{\prime }}}{z^{*}}-\frac{6}{r}\right]=\frac{2}{z^{*}}\left[\frac{1}{r^{2}}-8\pi \Pi^{\left(eff\right)}\right]. \end{array}$$

Integration of Equation (47) gives

(48) $$\begin{array}{c} e^{\nu (r)}=\frac{z^{*2}e^{\int \left(2z^{*}+\frac{4}{z^{*}r^{2}}\right)dr}}{r^{6}\left[2\int \left\{z^{*}\left(\frac{1-8\pi \Pi^{\left(eff\right)}r^{2}}{r^{8}}\right)e^{\int \left(2z^{*}+\frac{4}{z^{*}r^{2}}\right)dr}\right\}dr+{\bar{B}}_{1}^{*}\right]}. \end{array}$$

Any hyperbolically static symmetric solution can be outlined in detail with the support of two generating functions ($\Pi^{\left(eff\right)}$ and $z^{*}$), as shown by Equation (48). The corresponding structural variables of the locally anisotropic matter distributions become

(49) $$\begin{array}{cc} 4\pi \mu & =\frac{m^{\prime }f_{R}}{r^{2}}+\frac{1}{2}\left(\frac{f}{2}-\frac{Rf_{R}}{2}+e^{-\nu }f_{R}^{\prime \prime }-\frac{e^{-\nu }f_{R}^{\prime }\nu^{\prime }}{2}+\frac{2e^{-\nu }f_{R}^{\prime }}{r}\right), \end{array}$$

(50) $$\begin{array}{cc} 4\pi P_{r} & =f_{R}\left(\frac{z^{*}r\left(2m-r\right)-m+r}{r^{3}}\right)-\frac{1}{2}\left(-\frac{f}{2}+\frac{Rf_{R}}{2}-\frac{e^{-\nu }f_{R}^{\prime }\lambda^{\prime }}{2}+\frac{2e^{-\nu }f_{R}^{\prime }}{r}\right), \end{array}$$

(51) $$\begin{array}{cc} 8\pi P_{⊥} & =f_{R}\left\{\left(\frac{2mr-r^{2}}{r^{2}}\right)\left[z^{{*}^{\prime }}+\frac{1}{r^{2}}+z^{*2}-\frac{z^{*}}{r}\right]+z^{*}\left[\frac{m^{\prime }}{r}-\frac{m}{r^{2}}\right]\right\}-(\frac{Rf_{R}}{2}\\ & -\frac{f}{2}-\frac{e^{-\nu }f_{R}^{\prime }\lambda^{\prime }}{2}-e^{-\nu }f_{R}^{\prime \prime }+\frac{e^{-\nu }f_{R}^{\prime }\nu^{\prime }}{2}-\frac{e^{-\nu }f_{R}^{\prime }}{r}). \end{array}$$

Thus we have expressed the associated physical parameters of hyperbolically symmetric spacetime in terms of the auxiliary variables.

### 6.1. Conformally Flat Solutions

Due to the fact that the Weyl tensor plays a prominent role in the structure of fluid distribution, the exceptional case of ε=0 (conformal flatness) from Equations (20) and (29), could therefore be worth studying. Utilizing Equation (18) for ε=0 yields

(52) $$e^{-\nu -\lambda }\frac{\partial }{\partial r}\left[\frac{\lambda^{\prime }e^{\lambda }}{2r}\right]+\frac{\partial }{\partial r}\left[\frac{\lambda^{\prime }e^{-\nu }}{2r}\right]-\frac{\partial }{\partial r}\left[\frac{1+e^{-\nu }}{r^{2}}\right]=0.$$

Through the new variables, i.e., $\tilde{y}=e^{-\nu }$ and $\frac{\lambda^{\prime }}{2}=\frac{s^{\prime }}{s}$, Equation (52) becomes

(53) $${\tilde{y}}^{\prime }+2\left(\frac{s^{\prime \prime }-\frac{s^{\prime }}{r}+\frac{s}{r^{2}}}{s^{\prime }-\frac{w}{r}}\right)\tilde{y}+\frac{2s}{\left(s^{\prime }-\frac{s}{r}\right)r^{2}}=0,$$

The aforementioned equation upon integration generates the formal solution, which is given as follows

(54) $$\tilde{y}=e^{-\int h_{1}^{*}\left(r\right)dr}\left(\int e^{-\int h_{1}^{*}\left(r\right)dr}h_{2}^{*}\left(r\right)dr+{\bar{B}}_{2}^{*}\right),$$

here ${\bar{B}}_{2}^{*}$ indicates the integration constant and is defined as

$$\begin{array}{ccc} & & h_{1}^{*}\left(r\right)=2\frac{d}{dr}\left[ln\left(s^{\prime }-\frac{s}{r}\right)\right],\\ & & h_{2}^{*}\left(r\right)=\frac{-2s}{\left(s^{\prime }-\frac{s}{r}\right)r^{2}}. \end{array}$$

Feeding back the variables into their original values, Equation (54) becomes

(55) $$\frac{\lambda^{\prime }}{2}=\frac{e^{\nu /2}}{r}\sqrt{r^{2}e^{-\lambda }\beta^{*}-1}+\frac{1}{r}.$$

The junction conditions (both Darmois and Senovilla conditions) in f(R) gravity [61,62] provide

(56) $$\begin{array}{c} e^{\lambda_{\Sigma^{e}}}=\frac{2M}{r_{\Sigma }}-1,\;\;e^{\nu_{\Sigma^{e}}}={\left(\frac{2M}{r_{\Sigma }}-1\right)}^{-1},\;\;P_{r}^{\left(eff\right)}\left(r_{\Sigma^{e}}\right)=0. \end{array}$$

The value of $\beta^{*}$ is calculated as

$$\beta^{*}=\frac{9M^{2}-4Mr_{\Sigma^{e}}}{r_{\Sigma^{e}}^{4}}.$$

Integration of Equation (55) produces

$$e^{\lambda }=\beta^{*}r^{2}\sin^{2}\left(\int \frac{e^{\nu /2}}{r}dr+\alpha^{*}\right),$$

where α is an integration constant and can be found by applying the matching conditions discussed in Equation (56) as

$$\begin{array}{cc} \alpha^{*} & =\sin^{-1}\left[r_{\Sigma^{e}}{\left(\frac{\frac{2M}{r_{\Sigma^{e}}}-1}{9M^{2}-4r_{\Sigma_{e}}M}\right)}^{1/2}\right]-{\left[\int \frac{e^{\nu /2}}{r}dr\right]}_{\Sigma^{e}}. \end{array}$$

We have to impose an additional constraint in order to construct a particular model, as only one generating function can be determined using the conformal flatness condition. Therefore, we will consider the most extreme case, i.e., $P_{r}=0$ as an example. This solution is the hyperbolically symmetric counterpart of the model I for the spherically symmetric case, as studied by [63]. Equation (8) after putting $P_{r}=0$ produces

(57) $$\lambda^{\prime }=\frac{-\frac{1+e^{\nu }}{r}+\frac{re^{\nu }\chi_{1}\left(r\right)}{f_{R}}}{\chi_{2}\left(r\right)}.$$

where the values of $\chi_{1}\left(r\right)$ and $\chi_{2}\left(r\right)$ are defined in the Appendix A. Their values contain extra degrees of freedom due to metric f(R) gravity. Afterwards, substituting Equation (57) in (18) with the additional constraint of conformal flatness (ε=0) give

(58) $$\begin{array}{cc} \frac{{\left(1+e^{\nu }\right)}^{2}}{\chi_{2}\left(r\right)} & +4\left(1+e^{\nu }\right)\left[1+\frac{1}{\chi_{2}\left(r\right)}\right]+2\nu^{\prime }r\left[1+\frac{1}{\chi_{2}\left(r\right)}\right]+\frac{\nu^{\prime }re^{\nu }}{\chi_{2}\left(r\right)}\left[\frac{r^{2}\chi_{1}\left(r\right)}{f_{R}}-1\right]\\ & +\delta_{1}\left(r\right)=0, \end{array}$$

where the value of $\delta_{1}\left(r\right)$ is given in Appendix A. We will again achieve the aforementioned equation in GR if we substitute f(R)=R. Alternatively, Equation (58) on substitution of $e^{-\nu }=2g\left(r\right)-1$ gives

(59) $$\begin{array}{cc} \; & -rg^{\prime }\left[gf_{R}^{2}\chi_{2}\left(1+2\chi_{2}\right)-f_{R}^{2}\chi_{2}\left(1+\chi_{2}\right)+2r^{2}f_{R}\chi_{1}\chi_{2}\right]+g[gf_{R}^{2}\left(4+\chi_{2}^{2}+4\chi_{2}+2r\chi_{2}^{\prime }\right)\\ & -r^{2}f_{R}\chi_{1}-2f_{R}^{2}\chi_{2}-4r^{3}\chi_{1}\chi_{2}f_{R}^{\prime }-2f_{R}^{2}\chi_{2}^{2}+r^{3}f_{R}\chi_{1}^{\prime }\chi_{2}-rf_{R}^{2}\chi_{2}^{\prime }-r^{3}\chi_{1}\chi_{2}^{\prime }f_{R}]+\frac{\delta_{2}}{4}=0. \end{array}$$

where the value of $\delta_{2}$ is defined in Appendix A. Equation (59) upon integration produces

$$\begin{array}{cc} \; & B^{*}=g-\frac{1}{r[gf_{R}^{2}\chi_{2}\left(1+2\chi_{2}\right)-f_{R}^{2}\chi_{2}\left(1+\chi_{2}\right)+2r^{2}f_{R}\chi_{1}\chi_{2}]}(g[gf_{R}^{2}\left(4+\chi_{2}^{2}+4\chi_{2}+2r\chi_{2}^{\prime }\right)\\ & -r^{2}f_{R}\chi_{1}-2f_{R}^{2}\chi_{2}-4r^{3}\chi_{1}\chi_{2}f_{R}^{\prime }-2f_{R}^{2}\chi_{2}^{2}+r^{3}f_{R}\chi_{1}^{\prime }\chi_{2}-rf_{R}^{2}\chi_{2}^{\prime }-r^{3}\chi_{1}\chi_{2}^{\prime }f_{R}]+\frac{\delta_{2}}{4}) \end{array}$$

where $B^{*}$ is an integration constant. The combination of Equations (55) and (57) generate

$$e^{\lambda }=\frac{\beta^{*}r^{2}}{r^{2}\left(2g-1\right){\left[\frac{\frac{2g}{r(2g-1)}-\frac{r2g\chi_{1}}{f_{R}\left(2g-1\right)}}{2\chi_{2}}+\frac{1}{r}\right]}^{2}+1}.$$

We get the following results for the physical variables

(60) $$\begin{array}{cc} |\mu^{\left(eff\right)}| & =\frac{g\left(\delta_{3}+\delta_{4}\right)+\delta_{5}}{r^{2}\chi_{1}+2f_{R}\left(-1+g-\chi_{2}+2g\chi_{2}\right)}, \end{array}$$

(61) $$\begin{array}{cc} P_{⊥}^{\left(eff\right)} & =\frac{g^{3}\delta_{6}+g^{2}\delta_{7}+g\delta_{8}}{\left(2g-1\right)[r^{2}\chi_{1}+2f_{R}\left(-1+g-\chi_{2}+2g\chi_{2}\right)]}. \end{array}$$

The values of $\delta_{i}^{\prime }s$ where i=3,4,5,6,7,8 are given in the Appendix A. These values include the effects of f(R) terms. The two generating functions corresponding to this model are defined as

$$\begin{array}{cc} z= & \frac{-g+\frac{r^{2}\chi_{1}}{2f_{R}}+\left(2g-1\right)\chi_{2}}{r\left(2g-1\right)\chi_{2}}\\ \Pi^{\left(eff\right)}= & \frac{2\chi_{1}\chi_{2}+\frac{2gf_{R}^{\prime }}{r}-\frac{r\chi_{1}f_{R}^{\prime }}{f_{R}}}{16\pi \chi_{2}}-\frac{g^{3}\delta_{6}+g^{2}\delta_{7}+g\delta_{8}}{\left(2g-1\right)[r^{2}\chi_{1}+2f_{R}\left(-1+g-\chi_{2}+2g\chi_{2}\right)]} \end{array}$$

The value of $\Pi^{\left(eff\right)}$ is calculated using the equation $\Pi^{\left(eff\right)}=P_{r}^{\left(eff\right)}-P_{⊥}^{\left(eff\right)}$, which is subject to the constraint $P_{r}=0$.

### 6.2. A Model with Zero Complexity Factor

As previously stated, the scalar $Y_{TF}$ has been proven to be an appropriate measure of the complexity of the fluid distribution, as one can witness in the discussion from [54]. Therefore, finding a model (other than the homogeneous and isotropic solution) that meets the criterion of vanishing complexity $\left(Y_{TF}=0\right)$ would be intriguing. We must apply an extra limitation to achieve a specific model because there is an unlimited number of such solutions. Here, we will suppose the condition $P_{r}=0$ in addition to $Y_{TF}=0$. Substitution of $P_{r}=0$ in Equation (8) produces

(62) $$\lambda^{\prime }=\frac{\frac{-2g}{r}+\frac{r\chi_{1}}{f_{R}}}{\left(2g-1\right)\chi_{2}}$$

where $g=\frac{1+e^{-\nu }}{2}$. Applying the condition $\left(Y_{TF}=0\right)$ in Equation (44), it obtains that

(63) $$\begin{array}{cc} m_{T} & ={\left(m_{T}\right)}_{\Sigma^{e}}{\left(\frac{r}{r_{\Sigma e}}\right)}^{3}+\left(\frac{cosh\pi -1}{2}\right)r^{3}\int_{r}^{r_{\Sigma^{e}}}\frac{e^{\frac{\nu +\lambda }{2}}}{\tilde{r^{2}}}(-4\pi \Pi^{\left(eff\right)}\\ & -\frac{4\pi \Pi }{f_{R}}+\frac{1}{6f_{R}\left(K_{\pi }K_{\beta }+\frac{h_{\pi \beta }}{3}\right)}\left(h_{\pi \beta }V_{\xi }V^{\gamma }\nabla^{\xi }\nabla_{\gamma }f_{R}\right))d\tilde{r}. \end{array}$$

with the help of Equations (24), (62) and (63), and $g=\frac{1+e^{-\nu }}{2}$, we obtain

$$\begin{array}{cc} e^{\lambda } & =\frac{4\chi_{2}^{2}r^{4}\left(2g-1\right)}{r_{\Sigma }^{6}{\left(cosh\pi -1\right)}^{2}}{\left[\frac{2{\left(m_{T}\right)}_{\Sigma^{e}}+\left(Cosh\pi -1\right)r_{\Sigma^{e}}^{3}{\tilde{\chi }}_{3}}{-2g+\frac{r^{2}\chi_{1}}{f_{R}}}\right]}^{2}, \end{array}$$

where

$$\begin{array}{c} {\tilde{\chi }}_{3}=\int_{r}^{r_{\Sigma^{e}}}\frac{e^{\frac{\nu +\lambda }{2}}}{\tilde{r^{2}}}\left(-4\pi \Pi^{\left(eff\right)}-\frac{4\pi \Pi }{f_{R}}+\frac{1}{6f_{R}\left(K_{\pi }K_{\beta }+\frac{h_{\pi \beta }}{3}\right)}\left(h_{\pi \beta }V_{\xi }V^{\gamma }\nabla^{\xi }\nabla_{\gamma }f_{R}\right)\right)d\tilde{r}. \end{array}$$

Putting the zero complexity factor condition in Equation (41), it follows that

(64) $$\begin{array}{c} rg^{\prime }\left[f_{R}^{2}\chi_{2}\left(g-1\right)+\delta_{9}\right]+g\left[g\left(-f_{R}^{2}-4f_{R}^{2}\chi_{2}+\delta_{10}\right)+\delta_{11}\right]+\frac{\delta_{12}}{24}=0, \end{array}$$

where the values of the terms $\delta_{9},\;\delta_{10},\;\delta_{11}$ and $\delta_{12}$ are defined in Appendix A. These terms illustrate the effects of metric f(R) terms. With this background, the state determinants for this particular model are evaluated as

(65) $$\begin{array}{cc} |\mu^{\left(eff\right)}| & =\frac{g\left(\frac{g}{4f_{R}\pi r^{2}}+\frac{3gf_{R}}{4\pi r^{2}\chi_{2}}+\frac{2gf_{R}}{4\pi r^{2}}-\frac{2f_{R}}{4\pi r^{2}}+\delta_{13}\right)+\delta_{14}}{-2f_{R}+2gf_{R}+r^{2}\chi_{1}+r\chi_{2}f_{R}^{\prime }-2rg\chi_{2}f_{R}^{\prime }}, \end{array}$$

(66) $$\begin{array}{cc} P_{⊥}^{\left(eff\right)} & =\frac{g^{2}\left(-\frac{3f_{R}}{4\pi r^{2}}+\frac{3gf_{R}}{4\pi r^{2}}+\frac{3gf_{R}}{8\pi r^{2}\chi_{2}}+\delta_{15}\right)+g\delta_{16}}{2\left(2g-1\right)\left(gf_{R}-f_{R}+r^{2}\chi_{1}+r\chi_{2}f_{R}^{\prime }-2gr\chi_{2}f_{R}^{\prime }\right)}, \end{array}$$

where the values of the terms $\delta_{13},\;\delta_{14},\;\delta_{15}$ and $\delta_{16}$ represent the effects of the dark source terms, which are defined in Appendix A. The generating functions of this model are calculated as

$$\begin{array}{cc} \Pi^{\left(eff\right)} & =\frac{2\chi_{1}\chi_{2}+\frac{2gf_{R}^{\prime }}{r}-\frac{r\chi_{1}f_{R}^{\prime }}{f_{R}}}{16\pi \chi_{2}}-\frac{g^{2}\left(-\frac{3f_{R}}{4\pi r^{2}}+\frac{3gf_{R}}{4\pi r^{2}}+\frac{3gf_{R}}{8\pi r^{2}\chi_{2}}+\delta_{15}\right)+g\delta_{16}}{2\left(2g-1\right)\left(gf_{R}-f_{R}+r^{2}\chi_{1}+r\chi_{2}f_{R}^{\prime }-2gr\chi_{2}f_{R}^{\prime }\right)},\\ z & =\frac{-g+\frac{r^{2}\chi_{1}}{2f_{R}}+\left(2g-1\right)\chi_{2}}{r\left(2g-1\right)\chi_{2}}. \end{array}$$

All the results obtained for this model reduce to GR on substituting f(R)=R.

### 6.3. Stiff Equation of State

Finally, in this subsection we will look at a few solutions that fulfill the so-called stiff equation of state, which was initially presented by Zeldovich [64] and is expected to be convenient for illustrating ultradense matter. It presupposes that energy density equals pressure in its initial form. Here, we make an assumption

(67) $$\left|\mu \right|=P_{r}+\frac{1}{8\pi }\left(e^{-\nu }f_{R}^{\prime \prime }-\frac{e^{-\nu }f_{R}^{\prime }\left(\lambda^{\prime }+\nu^{\prime }\right)}{2}\right).$$

Equation (67), after inserting in Equation (15), produces

(68) $$\begin{array}{cc} \frac{\partial P_{r}}{\partial r}+\frac{2\Pi }{r} & +P_{r}\frac{\partial }{\partial r}\left(\frac{1}{f_{R}}\right)+\frac{1}{8\pi }\frac{\partial }{\partial r}\left(\frac{Rf_{R}}{2}-\frac{f}{2}-\frac{\lambda^{\prime }f_{R}^{\prime }e^{-\nu }}{2}-\frac{2f_{R}^{\prime }e^{-\nu }}{r}\right)\\ & +e^{-\nu }f_{R}^{\prime \prime }-\frac{e^{-\nu }f_{R}^{\prime }\nu^{\prime }}{2}-\frac{f_{R}^{\prime }e^{-\nu }}{r}=0. \end{array}$$

Now it can be observed that few additional information or constraints are needed to achieve particular solutions. Hence, we look at two specific cases as examples.

- When $P_{⊥}=0$
  Let us initially suppose that the tangential pressure does not exist. Then the integration of Equation (68) results in
  (69) $$\begin{array}{cc} P_{r}=\frac{A}{r^{2}} & +\frac{1}{r^{2}}\int r^{2}[P_{r}\frac{\partial }{\partial r}\left(\frac{1}{f_{R}}\right)+\frac{1}{8\pi }\frac{\partial }{\partial r}\left(\frac{Rf_{R}}{2}-\frac{f}{2}-\frac{\lambda^{\prime }f_{R}^{\prime }e^{-\nu }}{2}-\frac{2f_{R}^{\prime }e^{-\nu }}{r}\right)\\ & +e^{-\nu }f_{R}^{\prime \prime }-\frac{e^{-\nu }f_{R}^{\prime }\nu^{\prime }}{2}-\frac{f_{R}^{\prime }e^{-\nu }}{r}]dr,\;\;\\ \Rightarrow \left|\mu \right|=\frac{A}{r^{2}} & +\frac{1}{r^{2}}\int r^{2}[P_{r}\frac{\partial }{\partial r}\left(\frac{1}{f_{R}}\right)+\frac{1}{8\pi }\frac{\partial }{\partial r}\left(\frac{Rf_{R}}{2}-\frac{f}{2}-\frac{\lambda^{\prime }f_{R}^{\prime }e^{-\nu }}{2}-\frac{2f_{R}^{\prime }e^{-\nu }}{r}\right)\\ & +e^{-\nu }f_{R}^{\prime \prime }-\frac{e^{-\nu }f_{R}^{\prime }\nu^{\prime }}{2}-\frac{f_{R}^{\prime }e^{-\nu }}{r}]dr, \end{array}$$
  where *A* is the positive integration constant. When we combine Equations (11), (12) and (14) with Equation (69), the outcome is found as
  $$\begin{array}{c} m=4\pi Ar,\;\;e^{-\nu }=8\pi A-1,\;\;\lambda =constant. \end{array}$$
  Both the a.g.m. and the p.g.m.d. disappear in this model. There are no vanishing pressure surfaces for this solution, and the generating functions are
  (70) $$\begin{array}{c} \Pi =f_{R}\left[\frac{A}{r^{2}}-e^{-\nu }f_{R}^{\prime \prime }+\frac{e^{-\nu }f_{R}^{\prime }\nu^{\prime }}{2}+\frac{f_{R}^{\prime }e^{-\nu }}{r}\right],\;\;z^{*}=\frac{1}{r}. \end{array}$$
- When $Y_{TF}=0$
  This case satisfies stiff state equation along with $Y_{TF}=0$. In other words, we are considering less complex relativistic hyperbolical symmetric manifolds, whose energy density is specifically proportional to the pressure component. Therefore, we are clear to consider the simplest stiff fluid model (the one that meets the vanishing complexity factor criterion in addition to Equation (67)). Firstly, by implementing the former condition in Equation (43) and then feeding it back the resultant expression into Equation (68), we achieve
  (71) $$\frac{\partial^{2}P_{r}^{\left(eff\right)}}{\partial r^{2}}+\frac{2}{r}\left[\frac{\partial P_{r}^{\left(eff\right)}}{\partial r}\right]=\frac{\partial \chi_{4}}{\partial r}+\frac{4\chi_{4}}{r},$$
  where
  $$\begin{array}{cc} \chi_{4} & =\frac{2\Pi }{rf_{R}}-\frac{\chi_{3}}{12\pi rf_{R}},\\ \chi_{3} & =\frac{1}{\left(K_{\pi }K_{\beta }+\frac{h_{\pi \beta }}{3}\right)}\left(h_{\pi \beta }V_{\xi }V^{\gamma }\nabla^{\xi }\nabla_{\gamma }f_{R}\right). \end{array}$$
  The solution of Equation (71) is obtained as
  (72) $$P_{r}^{\left(eff\right)}=\frac{b}{r^{2}}-a+\int_{0}^{r}\left[\chi_{4}+\frac{2}{r^{2}}\int_{0}^{r}r\chi_{4}dr\right]dr,$$
  here *a* and *b* are two constants of integration, which are taken to be positive. With the support of Equations (11), (12) and (72) one can achieve
  (73) $$m=4\pi \left\{r\left(b-\frac{ar^{2}}{3}\right)+\int_{0}^{r}\int_{0}^{r}\left[\chi_{4}+\frac{2}{r^{2}}\int_{0}^{r}r\chi_{4}dr\right]drdr\right\}.$$
  One may calculate the fluid distribution by taking into consideration the surface $\Sigma^{e}$, which is restricted from the outside and specified as $r=r_{\Sigma^{e}}=constant$.
  (74) $$\begin{array}{cc} P_{r}^{\left(eff\right)} & =\int_{0}^{r}\left[\chi_{4}+\frac{2}{r^{2}}\int_{0}^{r}r\chi_{4}dr\right]dr-\int_{0}^{r_{\Sigma }}\left[\chi_{4}+\frac{2}{r^{2}}\int_{0}^{r}r\chi_{4}dr\right]{|}_{r=r_{\Sigma }}dr\\ \; & +b\left[\frac{1}{r^{2}}-\frac{1}{r_{\Sigma^{e}}^{2}}\right] \end{array}$$
  and
  (75) $$\begin{array}{cc} m & =4\pi \int_{0}^{r}\left[\chi_{4}+\frac{2}{r^{2}}\int_{0}^{r}r\chi_{4}dr\right]r^{2}dr+\frac{4\pi br}{3r_{\Sigma^{e}}^{2}}\left(3r_{\Sigma^{e}}^{2}-r^{2}\right)\\ & -4\pi r^{2}\int_{0}^{r_{\Sigma }}\left[\chi_{4}+\frac{2}{r^{2}}\int_{0}^{r}r\chi_{4}dr\right]{|}_{r=r_{\Sigma }}dr. \end{array}$$
  The following expression is produced from Equations (74) and (75) as
  $$\begin{array}{cc} \; & 4\pi P_{r}^{\left(eff\right)}r^{3}-m=-4\pi \int_{0}^{r}\left[\chi_{4}+\frac{2}{r^{2}}\int_{0}^{r}r\chi_{4}dr\right]r^{2}dr-\frac{8\pi br^{3}}{3r_{\Sigma^{e}}^{2}}\\ & +4\pi r^{2}\left(1-r\right)\int_{0}^{r_{\Sigma }}\left[\chi_{4}+\frac{2}{r^{2}}\int_{0}^{r}r\chi_{4}dr\right]dr{|}_{r=r_{\Sigma }}+4\pi r^{3}\int_{0}^{r}\left[\chi_{4}+\frac{2}{r^{2}}\int_{0}^{r}r\chi_{4}dr\right]r^{2}dr. \end{array}$$
  Finally, the $P_{⊥}^{eff}$ is determined as follows
  $$\begin{array}{cc} P_{⊥}^{\left(eff\right)} & =-\frac{b}{r_{\Sigma^{e}}^{2}}+\frac{r\chi_{4}}{2}+\int_{0}^{r}r\chi_{4}dr-\int_{0}^{r_{\Sigma }}\left[\chi_{4}+\frac{2}{r^{2}}\int_{0}^{r}r\chi_{4}dr\right]dr{|}_{r=r_{\Sigma }}\\ & +\int_{0}^{r}\left[\chi_{4}+\frac{2}{r^{2}}\int_{0}^{r}r\chi_{4}dr\right]dr. \end{array}$$

## 7. Conclusions

The rudimentary solutions of GR, such as the Schwarzschild and Kottler spherically symmetric exteriors are also solutions of the f(R) theories. The f(R) theory is an intriguing and reasonably straightforward alternative to GR. Here, we consider a static spacetime. Over and above, it would be ideal to have a static solution spanning the entire spacetime, based on the physically plausible viewpoint that any equilibrium ultimate state of a physical process should be static. The static, spherically symmetric, asymptotically flat, and empty exterior region is described by the Schwarzschild solution of the Einstein gravitational field equations. Therefore, outside the horizon, one has the standard Schwarzschild line element (e.g., where radius *r* of the self gravitating object is greater than two times the mass *m* of that object r>2m). However, it is widely known that no static observers can be defined inside the horizon. As a consequence, in order to obtain globally static solution the change in symmetry (and signature) is required. Otherwise, inside the horizon, static solution will not be possible to achieve (e.g., where radius *r* of the self gravitating object is lesser than two times the mass *m* of that object r<2m) as in [65,66].

The present work is aimed to analyze some characteristics of irrotational static hyperbolically symmetric objects. We performed this analysis under the correction of f(R) gravity, which permits some extra degrees of freedom that were not possible in GR. We assumed that the fluid has a different impact of pressure effects at different directions. For this, we looked at the entire spacetime continuum (0<r<∞). We preserve the temporal independence but adjust the spatial symmetry, rather than compromising the staticity in the region inside the horizon, i.e., r<2m [67]. The evaluation of the effective energy density reveals that it is inevitably negative, which is highly important in understanding various quantum field events because negative energies are strongly related to quantum field theory. The presence of dark source terms influences the tidal forces as well as the mass of a hyperbolically symmetric astronomical object. The repulsive aspect of the gravitational interaction as a result of the negative *a.g.m.* (if $4\pi P_{r}^{\left(eff\right)}r^{3}<m$) in the case of a fluid distribution was already highlighted in Equation (25).

Afterwards, various hyperbolically symmetric solutions accompanying two generating functions have been examined, specified with different models and constraints. In addition, the fluid cannot fill the area surrounding the center, implying that there is a cavity around the center that is empty. We have derived models whose equation looks quite similar in shape as that of GR, with the exception that their equations exhibit physical behavior that is influenced by the effective matter. The obtained results can be applicable to some physical systems as under:

- Our model is comprised of fluid having negative energy density. The presence of this property in the relativistic fluid suggests that our study could be applicable to various cosmological and astrophysical objects, such as wormholes, warp drive, etc. It is worthy to note that negative energies or energy density is compatible with quantum field theory;
- We found that a test particle moving over the hyperbolically symmetric objects cannot reach the central point of the symmetry. This is due to the formation of empty central vacuole. The existence of central vacuum cavity are often invoked in cosmological voids and haloes. Voids are underdense areas that spread within the cosmos to make large filaments. They are neither cylindrical nor spherical in shape;
- In addition to this, we have performed our study in f(R) theory. Depending upon the choice of the model, we could have above mentioned results at different cosmic eras, such as, phantom, dark energy, inflation, etc. Thus, due to our study, one can analyze the properties of hyperbolical anisotropic manifolds at different cosmic evolutionary stages;
- All of the results are compatible with GR findings when f(R)=R.

## Data Availability

All data generated or analyzed during this study are included in this published article.

## Appendix A

The values of $\mu^{\left(eff\right)}$, $P_{r}^{\left(eff\right)}$ and $P_{⊥}^{\left(eff\right)}$ that occurred in Equations (7)–(9) are given as

$$\begin{array}{cc} \mu^{\left(eff\right)} & =\frac{1}{f_{R}}\left[\mu +\frac{1}{8\pi }\left(\frac{f}{2}-\frac{Rf_{R}}{2}+e^{-\nu }f_{R}^{\prime \prime }-\frac{e^{-\nu }f_{R}^{\prime }\nu^{\prime }}{2}+\frac{2e^{-\nu }f_{R}^{\prime }}{r}\right)\right],\\ P_{r}^{\left(eff\right)} & =\frac{1}{f_{R}}\left[P_{r}+\frac{1}{8\pi }\left(-\frac{f}{2}+\frac{Rf_{R}}{2}-\frac{e^{-\nu }f_{R}^{\prime }\nu^{\prime }}{2}-\frac{2e^{-\nu }f_{R}^{\prime }}{r}\right)\right],\\ P_{⊥}^{\left(eff\right)} & =\frac{1}{f_{R}}[P_{⊥}+\frac{1}{8\pi }(-\frac{f}{2}+\frac{Rf_{R}}{2}-\frac{e^{-\nu }f_{R}^{\prime }\lambda^{\prime }}{2}-e^{-\nu }f_{R}^{\prime \prime }\\ & +\frac{e^{-\nu }f_{R}^{\prime }\nu^{\prime }}{2}-\frac{e^{-\nu }f_{R}^{\prime }}{r})]. \end{array}$$

The term $\xi^{DR}$ appeared in Equation (39) and in Equation (42) is defined as

$$\begin{array}{cc} \xi^{DR}= & \frac{1}{2f_{R}\left(K_{\pi }K_{\beta }+\frac{h_{\pi \beta }}{3}\right)}\left[h_{\pi }^{\xi }h_{\beta }^{\delta }\nabla^{\mu }\nabla_{\sigma }\epsilon_{\xi }^{\sigma \gamma }\epsilon_{\mu \gamma \delta }+\frac{2}{3}\nabla^{\mu }\nabla_{\sigma }h_{\mu }^{\sigma }h_{\pi \beta }\right]. \end{array}$$

The values of the terms $\chi_{1}\left(r\right)$ and $\chi_{2}\left(r\right)$ appeared in Equation (57) are

$$\begin{array}{cc} \chi_{1}\left(r\right) & =\frac{-f(r)}{2}+\frac{R\left(r\right)f_{R}\left(r\right)}{2}-\frac{2f_{R}^{\prime }e^{-\nu }}{r}\\ \chi_{2}\left(r\right) & =1+\frac{f_{R}^{\prime }}{rf_{R}} \end{array}$$

The term occurred in Equation (58) is calculated as

$$\begin{array}{cc} \delta_{1}\left(r\right) & =\frac{2e^{\nu }r^{3}}{f_{R}\chi_{2}\left(r\right)}\left[-\frac{\chi_{1}\left(r\right)f_{R}^{\prime }}{f_{R}}+\chi_{1}^{\prime }\left(r\right)-\frac{\chi_{1}\left(r\right)\chi_{2}^{\prime }\left(r\right)}{\chi_{2}\left(r\right)}\right]+\frac{2r\chi_{2}^{\prime }\left(r\right)}{\chi_{2}^{2}\left(r\right)}\left(1+e^{\nu }\right) \end{array}$$

The term $\delta_{2}$ appeared in Equation (59) is defined as

$$\begin{array}{cc} \delta_{2} & =r^{4}\chi_{1}^{2}+2r^{3}\chi_{1}\chi_{2}f_{R}^{\prime }-2r^{3}\chi_{1}^{\prime }\chi_{2}f_{R}+2r^{3}\chi_{1}\chi_{2}^{\prime }f_{R} \end{array}$$

The terms $\delta_{3}$, $\delta_{4}$ and $\delta_{5}$ occurred in Equation (60) is evaluated as

$$\begin{array}{cc} \delta_{3} & =\frac{5gf_{R}}{2\pi r^{2}}+\frac{gf_{R}}{2\pi r^{2}\chi_{2}}+\frac{3gf_{R}\chi_{2}}{\pi r^{2}}+\frac{gf_{R}\chi_{2}^{\prime }}{\pi r\chi_{2}}\\ \delta_{4} & =-\frac{3f_{R}}{2\pi r^{2}}+\frac{\chi_{1}}{4\pi }-\frac{\chi_{1}}{2\pi \chi_{2}}-\frac{3f_{R}\chi_{2}}{2\pi r^{2}}-\frac{r\chi_{1}f_{R}^{\prime }}{2\pi f_{R}}+\frac{r\chi_{1}^{\prime }}{2\pi }-\frac{f_{R}\chi_{2}^{\prime }}{2\pi r\chi_{2}}-\frac{r\chi_{1}\chi_{2}^{\prime }}{2\pi \chi_{2}}\\ \delta_{5} & =\frac{r^{2}\chi_{1}^{2}}{8\pi f_{R}\chi_{2}}+\frac{r\chi_{1}f_{R}^{\prime }}{4\pi f_{R}}-\frac{r\chi_{1}^{\prime }}{4\pi }+\frac{r\chi_{1}\chi_{2}^{\prime }}{2\pi \chi_{2}} \end{array}$$

The terms $\delta_{6}$, $\delta_{7}$ and $\delta_{8}$

$$\begin{array}{cc} \delta_{6}= & \frac{5f_{R}}{4\pi r^{2}}+\frac{f_{R}}{4\pi r^{2}\chi_{2}}+\frac{3f_{R}\chi_{2}}{2\pi r^{2}}+\frac{f_{R}\chi_{2}^{\prime }}{2\pi r\chi_{2}}\\ \delta_{7}= & -\frac{3f_{R}}{4\pi r^{2}}+\frac{\chi_{1}}{8\pi }-\frac{\chi_{1}}{4\pi \chi_{2}}-\frac{3f_{R}\chi_{2}}{4\pi r^{2}}+\frac{r\chi_{1}f_{R}^{\prime }}{4\pi f_{R}}+\frac{r\chi_{1}^{\prime }}{4\pi }-\frac{f_{R}\chi_{2}^{\prime }}{2\pi r\chi_{2}}-\frac{r\chi_{1}\chi_{2}^{\prime }}{4\pi \chi_{2}}\\ \delta_{8}= & \frac{r^{2}\chi_{1}^{2}}{16\pi f_{R}\chi_{2}}+\frac{r\chi_{1}f_{R}^{\prime }}{4\pi f_{R}}-\frac{r\chi_{1}^{\prime }}{8\pi }+\frac{r\chi_{1}\chi_{2}^{\prime }}{4\pi \chi_{2}} \end{array}$$

The terms $\delta_{9},\delta_{10},\delta_{11}$ and $\delta_{12}$ appeared in Equation (64) are evaluated as

$$\begin{array}{cc} \delta_{9}= & \frac{r^{2}f_{R}\chi_{1}\chi_{2}+rf_{R}\chi_{2}^{2}f_{R}^{\prime }}{2}-rgf_{R}f_{R}^{\prime }\chi_{2}^{2}\\ \delta_{10}= & 2rf_{R}\chi_{2}^{2}f_{R}^{\prime }-2rf_{R}^{2}\chi_{2}^{\prime }-2r^{2}f_{R}\chi_{2}^{2}f_{R}^{\prime \prime }\\ \delta_{11}= & 4f_{R}^{2}\chi_{2}+\frac{3r^{2}f_{R}\chi_{1}}{2}-\frac{r^{2}f_{R}\chi_{2}^{2}\chi_{3}}{3}+r^{2}\chi_{1}\chi_{2}f_{R}^{\prime }-4rf_{R}\chi_{2}^{2}f_{R}^{\prime }\\ & -r^{3}f_{R}\chi_{2}\chi_{1}^{\prime }+rf_{R}^{2}\chi_{2}^{\prime }+r^{3}f_{R}\chi_{1}\chi_{2}^{\prime }+4r^{2}f_{R}f_{R}^{\prime \prime }\chi_{2}^{2}\\ \delta_{12}= & -9r^{4}\chi_{1}^{2}+4r^{2}f_{R}\chi_{2}^{2}\chi_{3}-12r^{3}\chi_{1}\chi_{2}f_{R}^{\prime }+12rf_{R}\chi_{2}^{2}f_{R}^{\prime }\\ & +12r^{3}f_{R}\chi_{2}\chi_{1}^{\prime }-12r^{3}f_{R}\chi_{2}^{\prime }\chi_{1}-12r^{2}f_{R}\chi_{2}^{2}f_{R}^{\prime \prime } \end{array}$$

The terms $\delta_{13},\delta_{14}$ and $\delta_{15},\delta_{16}$ occurred in Equations (65) and (66) are calculated as

$$\begin{array}{cc} \delta_{13} & =-\frac{g\chi_{2}f_{R}^{\prime }}{\pi r}+\frac{gf_{R}\chi_{2}^{\prime }}{\pi r\chi_{2}}+\frac{g\chi_{2}f_{R}^{\prime \prime }}{\pi }-\frac{f_{R}}{\pi r^{2}}-\frac{3\chi_{1}}{4\pi \chi_{2}}-\frac{r\chi_{1}f_{R}^{\prime }}{2\pi f_{R}}+\frac{\chi_{2}f_{R}^{\prime }}{\pi r}+\frac{r\chi_{1}^{\prime }}{2\pi }\\ & -\frac{f_{R}\chi_{2}^{\prime }}{2\pi r\chi_{2}}-\frac{r\chi_{1}\chi_{2}^{\prime }}{2\pi \chi_{2}}-\frac{\chi_{2}f_{R}^{\prime \prime }}{\pi }+\frac{r^{2}\chi_{1}}{4\pi r^{2}}+\frac{r\chi_{2}f_{R}^{\prime }}{4\pi r^{2}}-\frac{2rg\chi_{2}f_{R}^{\prime }}{4\pi r^{2}}\\ \delta_{14} & =\frac{3r^{2}\chi_{1}^{2}}{16\pi f_{R}\chi_{2}}-\frac{\chi_{2}\chi_{3}}{12\pi }+\frac{r\chi_{1}f_{R}^{\prime }}{4\pi f_{R}}-\frac{\chi_{2}f_{R}^{\prime }}{4\pi r}-\frac{r\chi_{1}\chi_{2}^{\prime }}{4\pi \chi_{2}}+\frac{\chi_{2}f_{R}^{\prime \prime }}{4\pi }\\ \delta_{15} & =\frac{\chi_{1}}{8\pi }-\frac{3\chi_{1}}{8\pi \chi_{2}}+\frac{\chi_{2}\chi_{3}}{12\pi }-\frac{r\chi_{1}f_{R}^{\prime }}{4\pi f_{R}}+\frac{5\chi_{2}f_{R}^{\prime }}{8\pi r}-\frac{3g\chi_{2}f_{R}^{\prime }}{4\pi r}+\frac{r\chi_{1}^{\prime }}{4\pi }-\frac{f_{R}\chi_{2}^{\prime }}{4\pi r\chi_{2}}\\ & +\frac{gf_{R}\chi_{2}^{\prime }}{2\pi r\chi_{2}}-\frac{r\chi_{1}\chi_{2}^{\prime }}{4\pi \chi_{2}}-\frac{\chi_{2}f_{R}^{\prime \prime }}{2\pi }+\frac{g\chi_{2}f_{R}^{\prime \prime }}{2\pi }\\ \delta_{16} & =\frac{3r^{2}\chi_{1}^{2}}{32\pi f_{R}\chi_{2}}-\frac{\chi_{2}\chi_{3}}{24\pi }+\frac{r\chi_{1}f_{R}^{\prime }}{8\pi f_{R}}-\frac{\chi_{2}f_{R}^{\prime }}{8\pi r}-\frac{r\chi_{1}^{\prime }}{8\pi }+\frac{r\chi_{1}\chi_{2}^{\prime }}{8\pi \chi_{2}}+\frac{\chi_{2}f_{R}^{\prime \prime }}{8\pi } \end{array}$$
